# Exposure to multiple pathogens - serological evidence for Rift Valley fever virus, Coxiella burnetii, Bluetongue virus and Brucella spp. in cattle, sheep and goat in Mali

**DOI:** 10.1371/journal.pntd.0010342

**Published:** 2022-04-29

**Authors:** Michel Mainack Dione, Amadou Séry, Cheick Abou Kounta Sidibé, Barbara Wieland, Abdou Fall

**Affiliations:** 1 International Livestock Research Institute, Dakar, Senegal; 2 Laboratoire Central Vétérinaire, Bamako, Mali; 3 Institute of Virology and Immunology, Mittelhäusern, Switzerland; 4 Department of Infectious Diseases and Pathobiology, Vetsuisse Faculty, University of Bern, Bern, Switzerland; Baylor College of Medicine, UNITED STATES

## Abstract

An important problem for livestock production in Mali is occurrence of several infectious diseases. A particular challenge for control of pathogens that affect different species, especially in a system with mixed herds with cattle, sheep and goats. Therefore, this study aimed to investigate co-exposure with Rift Valley fever virus (RVFV), Coxiella burnetii, Bluetongue virus (BTV) and Brucella spp. in different livestock species in mixed herds. With the exception of BTV these pathogens are also zoonotic. A retrospective assessment was carried out on a biobank of sera of cattle and small ruminants collected from Sikasso and Mopti regions. Nine hundred and twelve samples from cattle (n = 304), sheep (n = 318) and goat (n = 290) were screened. Serology tests were conducted using commercial kits as per the protocol of the manufacturers. Sero-prevalence for RVFV was 12.8% (Confidence Interval 95%: 9.3–17.1%); 4.7% (2.7–7.7%) and 3.1% (1.4–5.8%) in cattle, sheep and goat respectively. For Coxiella burnetii, the sero-prevalence was 55.3% (49.5–60.9%), 22.6% (18.2–27.6%), and 16.9% (12.8–21.7%); in cattle, sheep and goat respectively; and for BTV sero-prevalence was 88.8% (84.72–92.13%), 51.6% (45.9–57.2%), 56.2% (50.3–62.0%) in cattle, sheep in goat respectively. Brucella sp. had the lowest sero-prevalence and was only detected in cattle and sheep. Regional differences were observed with sero-prevalence of Coxiella burnetii in sheep and goat with BTV in goat being significantly higher in Sikasso than in Mopti (p<0.001). Evidence of exposure to two pathogens in the same animal was most common for the combination Coxiella burnetii and BTV in cattle (51.6%), followed by sheep (17.0%) and goat (15.5%). Considering the scarcity of disease occurrence and epidemiological data in most sub-saharan countries including Mali, this multi-pathogen survey provides important evidence that cattle, sheep and goat are exposed to pathogens that may negatively impact productivity and pose a risk for public health. The results from this study highlight the urgent need for a better understanding of pathogen diversity and their impact on human and animal health in order to minimize resulting risks. Given that some of the pathogens investigated here are zoonotic, establishment of One-Health surveillance system to monitor disease in animals and people is warranted. Therefore, intersectoral collaboration is recommended.

## Author summary

High prevalence of Rift Valley fever virus, Coxiella burnetii and Bluetongue virus were observed in cattle, sheep, and goats. We provided evidence that animals of the same herds have to cope with several pathogens. This study points out to need for important follow up studies to fill further knowledge gaps related to impacts of livestock diseases in Mali, especially zoonoses, and how to address the challenges.

## Introduction

The livestock population of Mali ranks first among the West African Economic and Monetary Union (WAEMU) and second among the countries of the Economic Community of West African States (ECOWAS). Despite its strong potential and economic importance, livestock production faces many challenges. Indeed, the persistence of diseases, feed shortages and the lack of market opportunities for farmers are among the main constraints hindering the development of the sector [1]. Animal health challenges are dominated by high burden of infectious and parasitic pathologies [2]. Major disease control interventions such as vaccination only target key transboundary endemic diseases including peste des petits ruminants (PPR), ovine pasteurellosis or contagious bovine pleuro-pneumonia (CBPP). Little attention is given to non-reported diseases that also lower productivity and compromise public health such as Bluetongue, Rift Valley fever (RVF), Q fever and Brucellosis, which can affect different host species, for instance, cattle, sheep and goats. In addition, in case of zoonotic diseases, they represent a public health threat to communities that keep livestock, and knowledge about their prevalence and socio-economic impacts is lacking in most countries in sub-saharan Africa including Mali where they are endemic. Our study thus targeted the epidemiology of pathogens that have been under-reported in Mali and affect different species and may thus play an underestimated role in a system where mixed herds are common. The pathogens selected were Rift Valley fever virus (RVFV), Coxiella Burnetii, Bluetongue virus (BTV) and Brucella spp.

RVF is an acute arthropod-borne viral disease of domestic animals, such as buffalo, camels, cattle, goats, and sheep. Several different species of mosquitoes are competent vectors for the RVFV. Sharp rises in incidence of RVF most commonly occur after periods of heavy rainfall which lead to an abundance of mosquitoes. RVF is also an important zoonosis that can cause severe disease in humans. The disease in susceptible animals can vary in severity and is characterized by fever, listlessness, anorexia, disinclination to move, abortions, and high morbidity and mortality rates in neonatal animals [3]. A study carried out on cattle in two regions of Mali has reported a sero prevalence of RVFV of 2.4% in Sikasso and 3.6% in Mopti [4].

Q fever is a disease caused by the bacteria Coxiella burnetii. Domestic animals such as cattle, sheep and goats act as the major reservoirs of the virus which can infect a large variety of animals, humans, birds, and arthropods [5]. Human infection results from inhalation of contaminated aerosols, consumption of contaminated unpasteurized dairy products, direct contact with contaminated milk, urine, feces, or semen of infected animals, and tick bites [6]. It causes a mild disease in ruminants, but can cause abortions and still births in cattle, sheep and goats. Because it is highly infectious for humans, Q fever is an important zoonosis, with veterinarians, laboratory workers, farmers, and abattoir workers at particular risk. Surveys have shown that significant numbers of livestock handlers have antibodies indicating exposure to the virus [7]. Q fever is a major health constraint to productivity of small ruminants due to reproductive losses caused by abortions, stillbirth, infertility, orchitis amongst others as a result of infection [8]. In Mali, the disease is neglected in both human and livestock with no dedicated control strategy [9]. Coxiella burnetii sero-prevalence rates in small ruminants have been reported to be 21.5% in average in central, western and northern Mali between 2006 and 2009 [9].

Bluetongue is a vector-borne, viral disease affecting domestic and wild ruminants (primarily sheep and including cattle, goats, buffalo, antelope, deer, elk and camels) that is transmitted mainly by biting midges of the *Culicoides* species. The severity of disease varies among different species with symptoms being most severe in sheep resulting in deaths, weight loss and disruption in wool growth. In highly susceptible sheep, morbidity can be as high as 100%. Mortality ranges from 2–30% but can be as high as 70% [10]. Old studies carried out in 1992 in Mali have reported high sero-prevalence of bluetongue virus in sheep and goats (60.5%) [11].

Brucellosis is a well-known zoonosis that not only causes serious economic losses in the livestock, but also poses a permanent threat to public health. The disease affects cattle, swine, sheep and goats, camels, equines, and dogs. It may also infect other ruminants, some marine mammals, and humans. The disease in animals is characterized by abortions or otherwise reproductive failure. While animals typically recover, and will be able to have live offspring following the initial abortion, they may continue to shed the bacteria [12]. Transmission to human occurs through unprotected handling of tissues or body fluids from infected animals, consumption of un-pasteurized milk and milk products or through inhalation of Brucella-contaminated aerosols [13,14]. In Mali, most studies on Brucellosis in livestock have focused on cattle and milk. A study in Mopti reported a seroprevalence of Brucella melitensis of 58% and Brucella abortus of 49% in the human population in 2009 [15]. In 2017, an outbreak of bovine brucellosis was reported by the Directorate of Veterinary Services in Koulikoro and Ségou (report not published). The results obtained, although very limited, confirm the continued circulation of Brucella spp. in cattle and sheep in Mopti region.

Data on co-infections or evidence of exposure to several pathogens in cattle and small ruminants is very limited for sub-Saharian Africa, including Mali. Our study aims to address this knowledge gap by assessing previous exposure of sheep, goat and cattle to the selected pathogens in two regions of Mali (Sikasso and Mopti) representing different production systems.

## Methods

### Ethics statement

The samples used in this study were collected during a national disease surveillance activity. However, the authorization to test the samples for diseases other than those that were originally targeted by the surveillance system was given by the National Directorate of Veterinary Services (DNSV) ref n. 2021-0156-MDR-DNSV.

### Study site

The study was carried out in major livestock producing regions of Mali, namely Mopti (pastoral systems) and Sikasso (agropastoral system). The choice of the study area was dictated by the development project that supported the study namely the Feed the Future Mali Livestock Technology Scaling Program (FTF-MLTSP). The FTF-MLTSP strived to reduce the productivity gaps and expand the volume and value of ruminant livestock produced and marketed by 61,000 households in 31 FTF focus communes in Sikasso, Mopti and Timbuktu. To achieve this, FTF-MLTSP promoted the wide-scale dissemination of productivity enhancing technologies such as innovative animal health delivery systems that reduce disease burden in ruminant livestock practices coupled with innovative marketing strategies (https://www.ilri.org/research/projects/feed-future-mali-livestock-technology-scaling-program).

**Fig 1:**
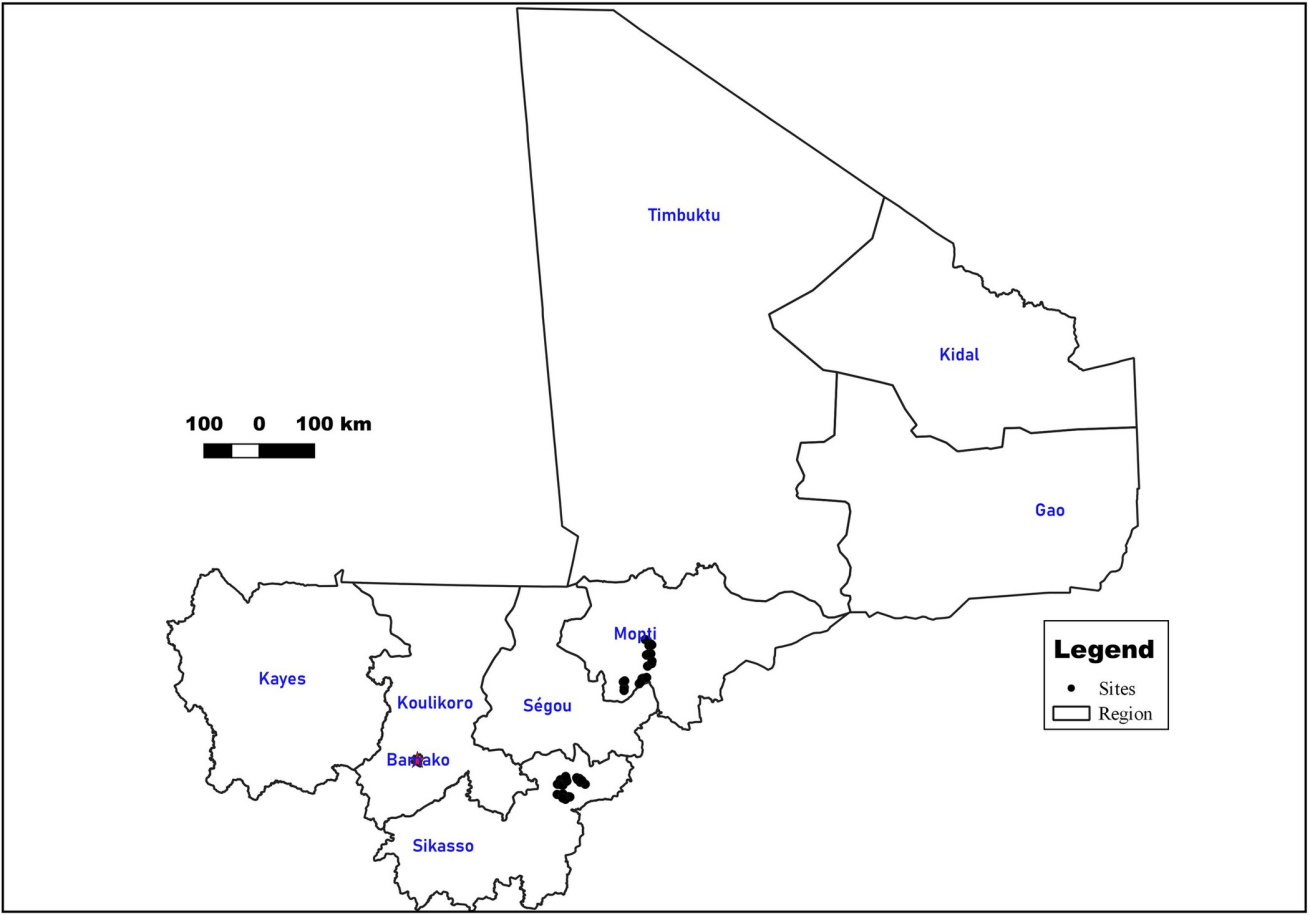
Map of Mali with the study sites. Mapped by Apollinaire Nombre of the Consultative Group on International Agricultural Research (CGIAR) using QGIS 3.4. Reproduced with permission.

In the Mopti region, reduced rainfall, overgrazing, expansion of grazing areas in crop land, drying of water points, and wind erosion are major constraints causing reduced feed availability. Therefore, pastoralists are forced to travel during part of the year to source feed for their animals. In contrast, the Sikasso region is among the wettest areas of Mali with a clear dominance of crop production over livestock farming. It is a system for which rangeland is the basic diet of animals. Access of farmers to veterinary services is easier in this region, compared to other regions. Sikasso region is also confronted with high occurrence of African Animal Trypanosomiasis [16] (Fig 1).

### Origin of the samples

Livestock development programs have supported the National Veterinary Services in implementing vaccination programs, including post-vaccination monitoring. This has yielded useful epidemiological data and sera samples for further studies. The FTF-MLSTP implemented in Sikasso and Mopti supported post-vaccination sero-surveillance of PPR and CBPP in the project communes in 2016 [17]. Four communes in each region were included in the post-vaccination sero-surveys. Proportional sampling of the villages was done in each commune. In Mopti, 20 villages were randomly selected, while in Sikasso 30 villages were selected. In each village, 3 herds were randomly selected and in each herd around 10 animals we enrolled for the post-vaccination sero-surveys. This resulted in 1,503 samples of cattle, and the same number for small ruminants (sheep and goats together). Serum samples were stored at the National Veterinary Laboratory in Bamako (Table 1).

**Table 1:** Sample size.

Region	Commune	Number of cattle during programme sero-monitoring	Sample size for cattle for this study	Number of sheep and goats during programme sero-monitoring	Sample size for sheep for this study	Sample size for goat for this study
Sikasso	Sinkolo	150	36	150	49	22
	Ngountjina	150	36	150	37	36
	Nafanga	151	52	151	36	36
	Zangasso	152	20	152	36	36
Total		603	144	603	158	130
Mopti	Socoura	210	40	210	40	40
	Sio	240	40	240	40	40
	Fakala	300	40	300	40	40
	Djenné	150	40	150	40	40
Total		900	160	900	160	160
Overall Total		1503	304	1503	318	290

### Sample size

To select sera for the screening, a sample size calculation was carried out using estimates of apparent sero-prevalence in Epi Info v7.2 (CDC). Considering an infinite population and an estimated Q fever sero-prevalence of 25% (average estimate at the higher end of published studies in Mali for Q fever) [9], an acceptable margin of error of 5%, a level of confidence of 95%, the sample size needed was a minimum of 289 animals per species (Table 1). Meta data including sex, species and region was collected. Serum samples were randomly selected from each commune of each district to ensure a representation of all animal species across all communes (Table 1).

### Laboratory procedure

Serology tests were conducted using commercial kits as per the protocol of the manufacturers. For RVFV, Rift Valley Fever Competition Multispecies ELISA (ID.vet Innovative Diagnostics, Montpellier, France) was used. With this test, both IgG and IgM are detected indistinguishably because competitive reactions are detected [18]. For Coxiella Burnetii, the Antibody Test Kit (IDEXX) was used [19], for BTV, Bluetongue Competition ELISA (ID.vet Innovative Diagnostics, Montpellier, France), a competitive ELISA kit which detected anti-VP7 antibodies, was used [20]. For Brucella sp., Rose Bengal Antigen for rapid slide agglutination test (ID.vet Innovative Diagnostics, Montpellier, France) was used [12].

### Data analysis

The data was entered into Microsoft Access then analyzed using STATA version 14 (Stata Corporation, 234 College Station, TX, USA). Tabulations and analytical statistics were used for data analysis. Chi-squared tests were carried out to compare sero-prevalence between species and regions.

## Results

### Sero-prevalence by pathogen and animal species

Cattle had a higher sero-prevalence for all pathogens compared to sheep and goat. Sero-prevalence for RVFV was 12.8%, 4.7% and 3.1% in cattle, sheep and goat respectively. Coxiella burnetii’s sero-prevalence was 55.3%, 22.6% and 16.9% in cattle, sheep and goat respectively, and Blutongue virus had a sero-prevalence of 88.8% in cattle, 56.2% in goat and 51.6% in sheep. Brucella sp. had the lowest sero-prevalence and was only detected in cattle (0.3%) and sheep (0.6%) with no significant difference between species (Table 2).

**Table 2:** Sero-prevalence at animal level.

Pathogen	Animal species (n)	Animal level seroprevalence with 95%CI	Pearson chi2 =	P-value
Rift Valley fever virus	Cattle	12.8% (39/304), 9.3–17.1	25.4742	0.000
	Sheep	4.7% (15/318), 2.7–7.7		
	Goat	3.1% (9/290), 1.4–5.8		
Coxiella burnetii	Cattle	55.3% (168/304), 49.5–60.9	119.3854	0.000
	Sheep	22.6% (72/318), 18.2–27.6		
	Goat	16.9% (49/290), 12.8–21.7		
Blutongue virus	Cattle	88.8% (270/304), 84.7–92.1	111.4527	0.000
	Sheep	51.6% (164/318), 45.9–57.2		
	Goat	56.2% (163/290), 50.3–62.0		
Brucella sp.	Cattle	0.3% (1/304), 0.0–1.8	1.8299	0.401
	Sheep	0.63% (2/318), 0.1–2.2		
	Goat	0.00		

### Sero-prevalence by animal species and by region

There was no significant difference in pathogen exposure in cattle across regions. Sero-prevalence of Coxiella burnetii in sheep was significantly higher in Sikasso compared to Mopti (32.9% and 13.1% respectively). The same trend was observed in goat (23.8% and 11.2% respectively). Sero-prevalence of BTV in goat was also significantly higher in Sikasso compared to Mopti (66.9% and 47.5% respectively). No significant differences were observed for RVFV, BTV and Brucella sp. in cattle (Table 3).

**Table 3:** Sero-prevalence by animal species and by region.

Species	Pathogen	Region (n)	Animal level seroprevalence with 95% CI	Pearson chi2	P-value
Cattle	Rift Valley fever virus	Mopti	15.6% (25/160), 10.4–22.2	2.3613	0.124
		Sikasso	9.7% (14/144), 5.4–15.8		
	Coxiella burnetii	Mopti	55.0% (88/160), 46.9–62.9	0.0095	0.923
		Sikasso	55.6% (80/144), 47.0–63.8		
	Blutongue virus	Mopti	90.6% (145/160), 85.0–94.7	1.1130	0.291
		Sikasso	86.8% (125/144), 80.2–91.9		
	Brucella sp.	Mopti	0.6% (1/160), 0.02–3.4	0.9030	0.342
		Sikasso	0.0 (0/130)		
Sheep	Rift valley fever virus	Mopti	2.5% (4/160), 0.69–6.28	3.5216	0.061
		Sikasso	7.0% (11/158), 3.5–12.1		
	Coxiella burnetii	Mopti	13.1% (21/160), 8.3–19.4	16.6507	0.000
		Sikasso	32.3% (51/158), 25.1–40.2		
	Blutongue virus	Mopti	56.9% (91/160), 48.8–64.7	3.6255	0.057
		Sikasso	46.2% (73/158), 38.2–54.3		
	Brucella sp.	Mopti	1.2% (2/160), 0.15–4.4	1.9875	0.159
		Sikasso	0.0 (0/130)		
Goat	Rift Valley fever virus	Mopti	1.9% (3/160), 0.4–5.4	1.7912	0.181
		Sikasso	4.6% (6/130), 1.7–9.8		
	Coxiella burnetii	Mopti	11.2% (18/160), 6.8–17.2	8.1045	0.004
		Sikasso	23.8% (31/130), 16.8–32.1		
	Blutongue virus	Mopti	47.5% (76/160), 39.6–55.5	10.9927	0.001
		Sikasso	66.9% (87/130), 58.1–74.9		
	Brucella sp.	Mopti	0 (0/160)	-	-
		Sikasso	0 (0/130)		

### Exposure to several pathogens

**Fig 2:**
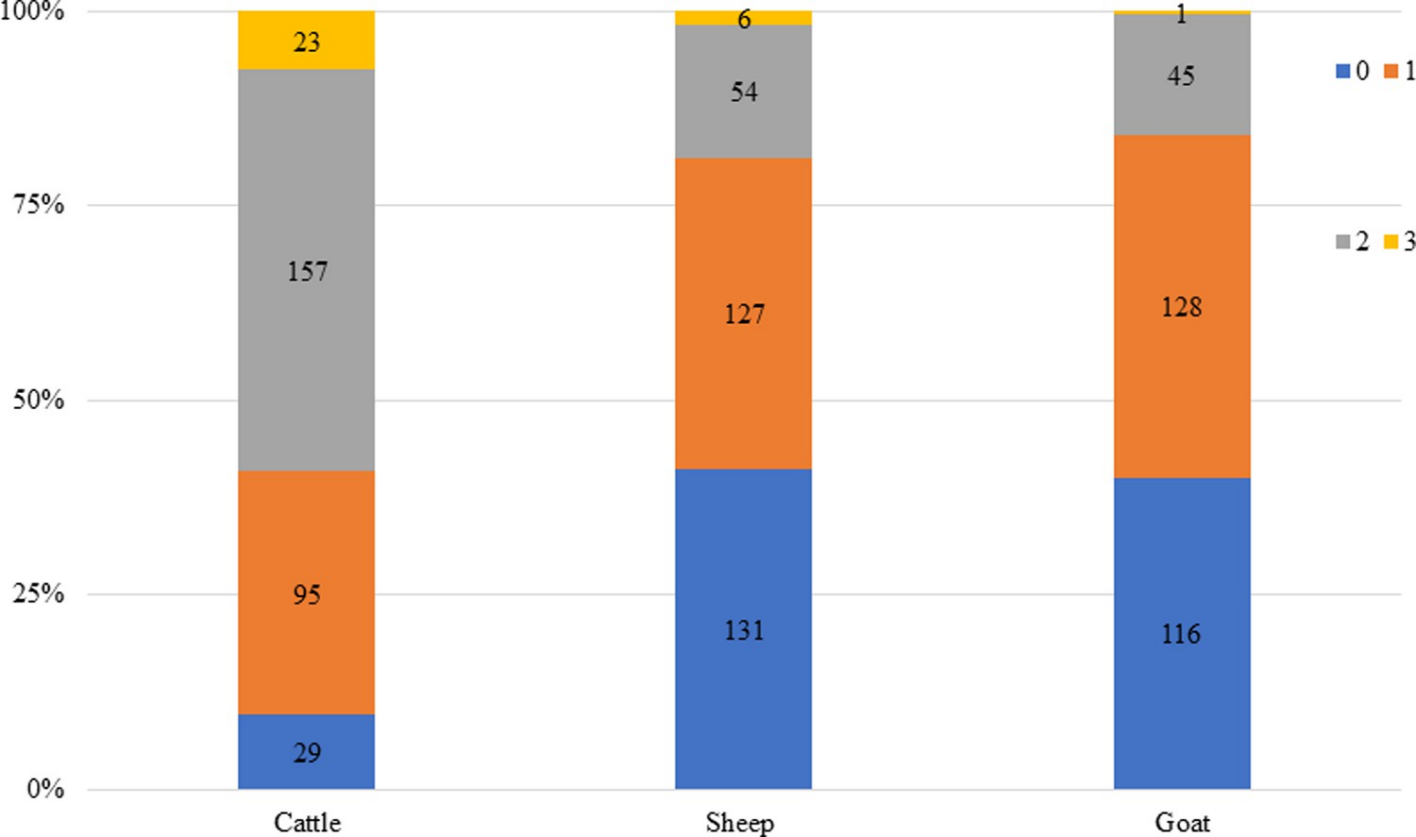
Sero-positivity profiles of co-infections. 0 (blue): single infection; 1 (pink): co-infection with one pathogen; 2 (grey): co-infection with two pathogens. 3 (orange): co-infection with three pathogens.

Serological profiles of examined samples showed seropositivity for two pathogens were most common with Coxiella burnetii and BTV in cattle (51.6%), followed by sheep (17.0%) and goat (15.5%). Previous exposure to three pathogens was observed for the combination of Coxiella burnetii, RVFV virus and BTV and was highest in cattle with an average of 7.6%, followed by sheep (1.9%) and goat (0.34%) (Fig 2).

## Discussion

This study provides for Mali data on single and multiple exposure of different livestock species including cattle, sheep, and goat to four important pathogens namely RVFV, Coxiella burnetii, Bluetongue virus and Brucella spp. These pathogens, although considered as important are not under surveillance in Mali.

Sero-prevalences rates of RVFV in cattle reported in our study (9.7% in Sikasso and 15.6% in Mopti) are higher than those reported in previous studies in cattle (2.4% in Sikasso and 3.6% in Mopti) [4]. Cattle serving as significant amplifier of RVFV [21], could play an important role in the maintenance of the virus in the in mixed herds. The increase in the prevalence in cattle and the reporting of human cases of RVFV infection in Malians in 2016 [22] indicates the need to keep this disease under closer surveillance in the future. Therefore, a high-performance surveillance system is required for early detection of RVF outbreaks to avoid the economic losses and possible zoonotic infection of human that can result from emergence of the disease [23]. Such surveillance system should be risk-based, for example during times when RVF is likely to occur because of presence of the vector. It was not surprising that the prevalence of RVFV was higher in Sikasso where vectors can survive better because if the prevailing climatic environment, therefore, more research on the vector is also recommended.

Coxiella burnetii sero-prevalence rates were significantly higher in sheep (32.3%) and goat (23.8%) in Sikasso compared to Mopti (13.1% in sheep and 11.2% in goat). These sero-prevalence rates are higher than those reported in previous studies carried out between 2006 and 2009 in central (10.8%), western and northern (5.8%) Mali [9]. In Africa, the highest seropositivity rates of Coxiella burnetii were reported from areas with the highest density of livestock (>100 per 100 inhabitants) and these included Mali, Burkina Faso, Nigeria and Central African Republic [24]. Sikasso region being an agro-pastoral production system, farmers have adopted a more sedentary system with animal density being higher than in Mopti, resulting into people living closer to their animals. This could explain the higher rate of seropositivity to Coxiella burnetii found in Sikasso. Coxiella burnetii reported among febrile urban patients in Mali point to an indication of the existence of a potential animal to human transmission cycle [25]. Given the risk of exposure of Coxiella burnetii to humans, a neglect of the disease among livestock farming communities could endanger the lives of those who work and live in close proximity to the livestock farms [26].

Sero-prevalence rates of BTV reported in our study are comparable to previous studies that have been reported in small ruminants in Mali (60.5% in average in sheep and goat) [11]. However, the sero-prevalence of BTV in cattle (88.8%) is significantly higher compared to sheep (51.6%) and goat (56.2%) confirming existing literature reporting that cattle often have a higher infection rate than sheep and demonstration and severity of clinical signs varies depending on the strain of virus [10]. Our study has also reported significant higher sero-prevalence of BTV in goat in Sikasso compared to Mopti. This could be explained by the fact that the Sikasso climate is more humid, and its environment is more conducive for the vector (Culicoides) habitat. Studies on the small-scale movement of Culicoides between farms and adjacent wildlife habitats, as well as on the frequency of contact between livestock and wildlife are needed to better understand BTV ecology [27].

Detection of antibodies against Brucella spp. although very limited in this study (0.3% in cattle, 0.63% in sheep and 0% in goat), confirm the continued circulation of Brucella spp. in cattle and sheep in Mopti region. These results are lower than those reported in sheep and goats in a recent study in Mali (4.1%) [28]. Reported seroprevalence of Brucella melitensis of 58% and Brucella abortus of 49% in the human population in 2009 is an indication of high risk of the disease to human [15]. The surveillance of brucellosis in humans is complicated by the un-availability of accurate diagnostic tools and the low knowledge of the medical professionals of the disease.

The observation that overall sero-prevalence of RVFV, Coxiella burnetii and BTV are significantly higher in cattle compared to small ruminants can be explained by the fact that cattle stays in the herd much longer than small ruminants the shorter-cycle, and thus are more likely to have been exposed for longer duration to the pathogens in the past. Not surprisingly, this study provided evidence that a single animal is exposed to different pathogens, which means that mixed infections seem common in livestock in Mali. Whether infections occur simultaneously or not cannot be concluded from our data, but given the high levels of prevalence, co-infections seem likely. For sure our data suggest that animals are continuously exposed to a range of pathogens, especially as the studied diseases are not included in any control programs. Given the poor biosecurity on smallholder livestock farms, it is expected that co-infections will remain and will continue to contribute to lowering immunity of livestock, hence exposing animals to various diseases. Since there are no vaccination programmes against the studied diseases in Mali, these specific sero-prevalences can be attributed to natural exposure to pathogens. The increasing sero-prevalence rates observed for RVFV, Coxiella burnetii and BTV compared to studies in the past, point out to a possibility of increasing risk to animal and human. Indeed, co-infection with zoonotic and other pathogens is likely to be a frequent occurrence in poor communities in tropical and sub-tropical Africa, imposing a combined but typically unquantified burden. Such communities may be coping with a wide range of endemic infectious diseases in both people and their animals [29]. The findings of our study highlight the urgent need to explore in an in-depth manner complex health issues at the interface of human and animals in pastoral and agro-pastoral livestock production systems in Mali. For this it is important not to focus on a single disease in a single species only, but to address the problem as a system of different species having to cope with several pathogens. Also needed are environmental including entomological studies, given that some of the pathogens are vector-borne. This situation also indicates that an integrated surveillance system through a One Health approach would be a huge step in the right direction to tackling livestock diseases and protect human health.

Serological surveys provide invaluable insight into the natural history and epidemiology of infection and can be used to estimate the burden of diseases particularly in developing countries where diagnostic capacities are limited [30]. Serologic positivity being a proxy for infection, may not accurately reflect actual infection rates, making it difficult to assess the exact time at which an animal acquired an infection. However, in the absence of vaccination, the presence of antibodies against these pathogens in the serum collected in his study clearly indicates that animals of the surveyed regions have already been infected by the pathogens that have been circulating in the herd.

## Conclusion

High prevalence of RVFV, Coxiella burnetii and BTV were observed in cattle, sheep, and goats, hence providing evidence that animals have to cope with several pathogens. This study points out to the need for important follow up studies to fill further knowledge gaps related to impacts of livestock diseases in Mali, especially zoonoses, and how to address the challenges. Interventions to address zoonoses, including vector borne diseases, should use a One-Heath approach to establish the risk factors for their occurrence in a particular setting at a system level; assess the knowledge, attitudes, practices of livestock owners; and carry out socio-economic studies to better estimate their impact on public health and on the livelihoods of farmers. For control program it will be key to operate at herd level including all present species since these diseases occur in different species at the same time. Therefore, disease surveillance and monitoring systems that consider both human and animals should be considered by the government as such a combination would result in an added value through reduced disease occurrence in livestock and better public health thanks to reduced zoonoses risks.

## Supporting information

S1 Data Sero-prevalence data by region and animal species. (XLSX)
